# Primary neuroendocrine tumor of the pineal gland: a case report

**DOI:** 10.1186/s12883-021-02351-0

**Published:** 2021-08-20

**Authors:** Angela Cheng, Jane Barron, Oliver Holmes, Peter Bartlett, Gregory Jenkins, Melanie Seal

**Affiliations:** 1 Faculty of Medicine, Memorial University, 105-78 Thorburn Road, St. John’s, NL A1B3T4 Canada; 2 Discipline of Laboratory Medicine (Neuropathology), Memorial University Faculty of Medicine, St. John’s, Canada; 3 Discipline of Oncology (Radiation Oncology), Memorial University Faculty of Medicine, St. John’s, Canada; 4 Department of Radiology, Memorial University Faculty of Medicine, St. John’s, Canada; 5 Department of Surgery (Neurosurgery), Memorial University Faculty of Medicine, St. John’s, Canada; 6 Discipline of Oncology (Medical Oncology), Memorial University Faculty of Medicine, St. John’s, Canada

**Keywords:** Neuroendocrine carcinoma, Pineal gland, Case report

## Abstract

**Introduction:**

Primary intracranial neuroendocrine tumors are exceedingly rare, with few cases in the literature. We present a case of a primary neuroendocrine carcinoma of the pineal gland, which is the second that has ever been reported.

**Case presentation:**

A 53-year-old male patient presented with vomiting, weakness, and headaches. Imaging revealed a lesion in the pineal region, which was surgically resected. This mass was characterized by histology as a neuroendocrine carcinoma, given the presence of neuroendocrine markers and cytokeratin markers with absence of a primary lesion elsewhere on imaging.

**Conclusions:**

There are currently no guidelines on the management of primary intracranial neuroendocrine tumors. In this case, the patient underwent surgical resection and craniospinal radiotherapy. He subsequently received one cycle of chemotherapy with temozolomide, an alkylating agent, but he unfortunately did not tolerate treatment. A multidisciplinary decision was made along with the patient and his family to focus on palliative care. Eighteen months after the initial presentation, disease recurred in the patient’s neck. The patient underwent resection to control the metastases, with a plan to follow with radiotherapy and chemotherapy. Unfortunately, the patient became unwell and died at 21 months after initial diagnosis. This demonstrates a need for continued research and reporting on this uncommon disease entity.

## Introduction

Neuroendocrine tumors (NETs) are a heterogeneous and uncommon group of tumors. They are composed of cells that contain dense core granules, hence the prefix ‘neuro’, and have secretory abilities, described by the term ‘endocrine’. While circulating biomarkers such as chromogranin A (CgA) and neuron specific enolase (NSE) can increase with the presence of a NET, the measurement of these peptides is not recommended for screening as they are unspecific markers and can be elevated due to other clinical conditions. For instance, CgA can be elevated in patients that are taking a proton pump inhibitor or in those with renal insufficiency, while NSE can be raised due to the presence of small-cell lung carcinoma [1]. Therefore, diagnosis requires tissue biopsy and the presence of neuroendocrine markers such as CgA, NSE, and synaptophysin on histological examination. The most common anatomical origins of these tumors are along the gastrointestinal tract and within the bronchopulmonary segments. Rarely, these tumors can occur intracranially, with nine reported cases of primary origin in this site [2–9]. Pineal gland origin is even less common, with only one previous case having been reported [2]. Pineal region tumors, given their proximity to the cerebral aqueduct, can lead to obstruction of cerebrospinal fluid (CSF) outflow, resulting in downstream effects such as hydrocephalus. To our knowledge, this is the second case that has been reported of a neuroendocrine carcinoma originating in the pineal region.

## Case presentation

### Clinical summary

**Fig. 1:**
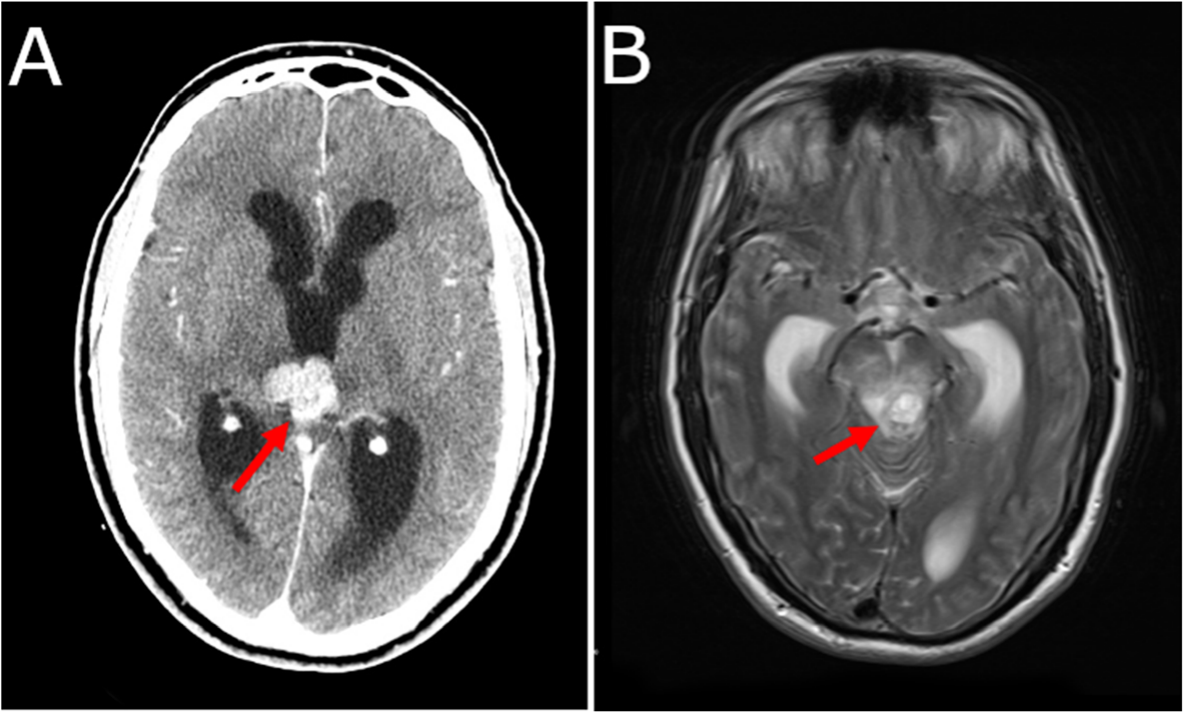
Pre-operative axial radiological images of the brain. **A** Computed tomography scan demonstrating the neoplasm in the pineal region with central calcification. **B** Magnetic resonance imaging scan demonstrating the neoplasm within the pineal region

A previously healthy 53-year-old male was admitted to hospital after presenting with a five-day history of intractable vomiting, generalized weakness, and ataxia. Additionally, he reported that he had been experiencing severe headaches. The patient had an unremarkable medical history, with hypertension as his only medical condition. On physical examination, he appeared lethargic and was frequently retching and vomiting. The only neurological findings were bilateral papilledema and impaired upward and downward gaze. Due to the patient’s condition, his gait could not be safely assessed. Computed tomography (CT) and magnetic resonance imaging (MRI) scans revealed hydrocephalus secondary to an outflow obstruction of the third ventricle. The obstructing mass measured 2.5 cm × 3 cm × 3.2 cm and appeared hyperdense, demonstrating enhancement with contrast. It was centred in the pineal region and had central calcification. There was mass effect on the adjacent midbrain. The midbrain was edematous, possibly indicating invasion. The remainder of the brain appeared normal (Fig. 1A, B).

The patient underwent endoscopic biopsy and endoscopic third ventriculostomy (ETV). An external ventricular drain (EVD) was placed due to moderate tumor bleeding during the biopsy. This drain was then weaned and removed on post-operation day three after a CT head showed improvement in the patient’s hydrocephalus. After the biopsy was reported as a malignancy, we offered the patient surgical resection of the tumor. We performed a craniotomy through the occipital bone. A supracerebellar, infratentorial approach was used to achieve a subtotal resection.

The patient developed a communicating hydrocephalus while recovering after the resection, requiring an EVD. The EVD became blocked twice, requiring reinsertion of the drain. The second time the drain blocked, the patient became drowsy, prompting us to place a permanent ventriculoperitoneal shunt. A ventriculoperitoneal shunt was not placed during the resection surgery as the non-communicating hydrocephalus was adequately treated with the ETV.

Histological analysis of the biopsied and resected tumor tissues presented a diagnostic challenge, given the rarity of primary neuroendocrine tumors of the pineal gland. The initial differential diagnosis included pineal parenchymal tumors, such as a pineoblastoma, as well as metastatic neuroendocrine carcinoma. The tissue was evaluated by four external pathologists, as well as a local Neuropathologist, and diagnosed as a neuroendocrine carcinoma based on the cytokeratin and synaptophysin positivity. Positron emission tomography-computed tomography (PET/CT) demonstrated fluorodeoxyglucose (FDG) avidity in the pineal gland with no evidence of another primary site, strongly suggesting that this was a primary neuroendocrine carcinoma of the pineal gland. It is worth mentioning that PET/CT revealed a small incidentaloma –without FDG avidity – in the upper pole of the left kidney suggestive of renal cell carcinoma (RCC).

During his hospitalization, the patient was found to have hypercalcemia and elevated parathyroid hormone (PTH). Consultation with an Endocrinologist revealed that he had primary hyperparathyroidism. Two months after his initial presentation, the patient had lost 24 kg, and was unable to speak or sit up in bed but retained the ability to move all limbs independently. At that time, the patient’s performance status was Eastern Cooperative Oncology Group (ECOG) 4 and he was not a candidate for further therapy. After discussions with the patient and his family, the patient was transferred home for palliative care.

Over the subsequent months, the patient’s condition improved significantly. He was reassessed and treated with craniospinal radiation in two phases (5400 cGy in 30 fractions to the residual enhancing tumor and resection cavity, and 3600 cGy in 20 fractions to the craniospinal axis). Post-radiation, the plan was to administer systemic therapy with palliative intent to slow disease progression and maintain quality of life. Approximately 8 months after his initial presentation, the patient’s performance status improved to ECOG-2 and he received his first cycle of chemotherapy (temozolomide 150 mg/m2 given orally daily for 5 days of a planned 28-day cycle). Unfortunately, he did not tolerate the drug well and was admitted to hospital for 5 weeks. Systemic therapy was discontinued. The patient recovered at home, and it was decided, in consultation with the patient and family, that surveillance would be the preferred approach.

**Fig. 2:**
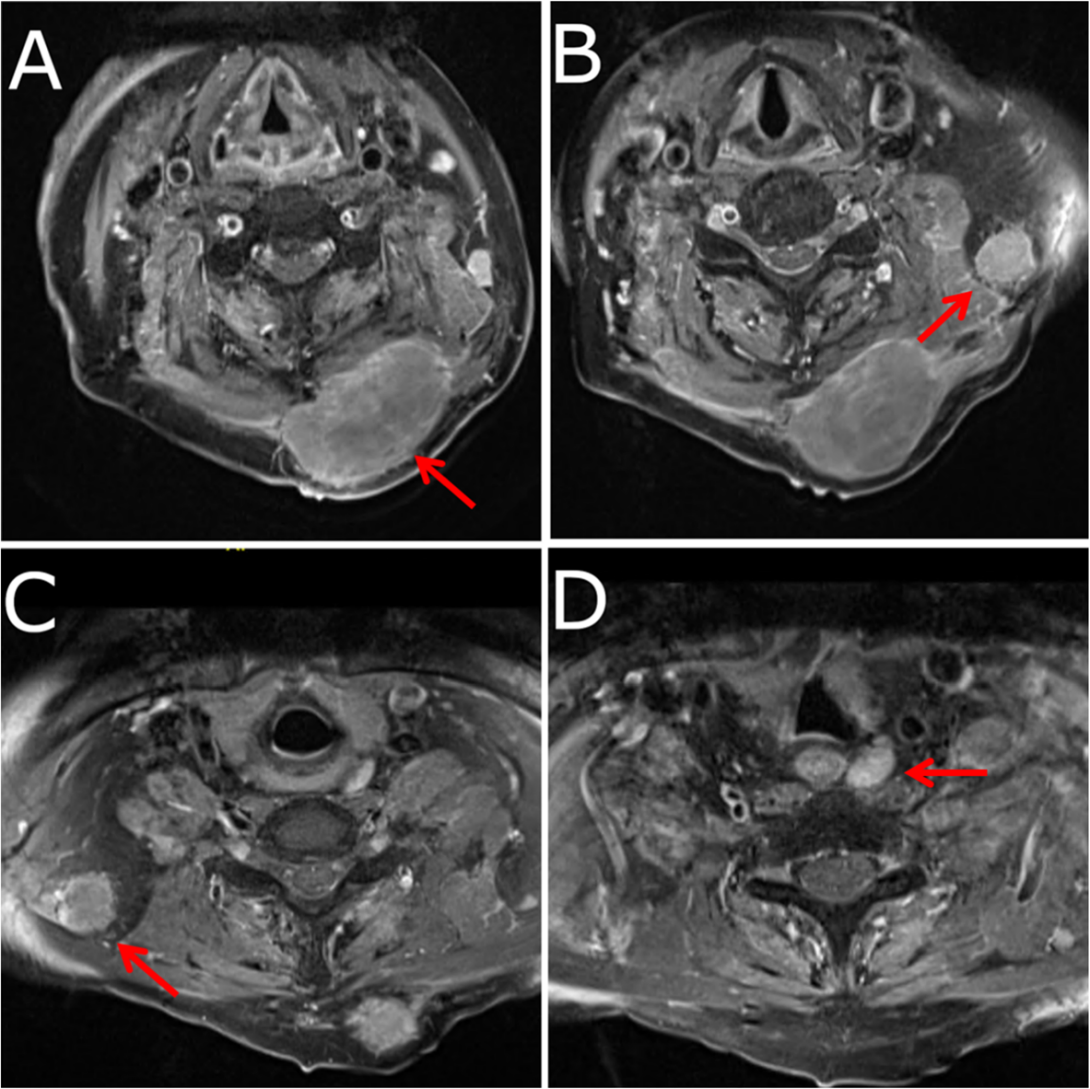
Four magnetic resonance images demonstrating metastatic disease throughout the soft tissue of the patient’s neck. **A** The clinically palpable lesion is shown located left of the midline of the posterior neck. **B** A lesion is shown located in the left posterior neck at level Vb. **C** A lesion is shown located in the right posterior neck at level Vb. **D** A lesion is shown located on the left side of the paraesophageal region at level VI

Eighteen months after his initial presentation, the patient developed a rapidly growing lesion on his posterior neck that tripled in size over 2 months. MRI showed at least four enhancing lesions in the soft tissue of the neck (Fig. 2). The lesion that was clinically palpable measured 56 mm × 35 mm × 55 mm and was located left of the midline of the posterior neck (Fig. 2A). The second lesion was in the left posterior neck at level Vb, measuring 16 mm × 17 mm × 17 mm (Fig. 2B). The third lesion was located on the right posterior neck at level Vb, measuring 22 mm × 18 mm × 20 mm (Fig. 2C). The fourth lesion was paraesophageal on the left at level VI, measuring 10 mm × 18 mm (Fig. 2D). The smaller lesions were likely lymph node metastases. A biopsy of the largest lesion was performed, and histology was highly suggestive of a metastatic lesion from the original pineal tumor. The treatment plan at this time was local resection, followed by radiation to control the metastases and potential platinum-based chemotherapy. The clinically palpable neck mass was surgically excised from the soft tissue 3 months after it was initially identified. Unfortunately, the patient became unwell prior to starting radiation and died 21 months after his initial diagnosis (Table 1).

**Table 1:** Timeline showcasing the relevant events from episode of care

Past medical history: Hypertension			
Date	Summary	Diagnostic Testing	Intervention
January 2018	Patient presented with five-day history of vomiting, weakness, balance difficulties, and headaches.	CT head: aggressive pineal tumor with hydrocephalus.	Started on IV steroids and transported to tertiary care centre.
January 2018	Patient experiencing symptoms of hydrocephalus.		Third ventriculostomy, biopsy of the pineal tumor, and insertion of external ventricular drain and ICP monitor.
February 2018	Patient experienced minimal symptom relief from drain placement.		Surgical subtotal resection of the pineal tumor for symptom relief.
March 2018	Patient continued to experience symptoms from hydrocephalus.	CT head: hydrocephalus.	Ventriculoperitoneal shunt placed.
March 2018	Patient assessed by Medical Oncology and Radiation Oncology. The patient was ECOG-4 at that time. Incidental hyperparathyroidism and hypercalcemia discovered after Endocrinology consultation.	PET-CT: no evidence of FDG-avid neoplasia elsewhere in body. Small renal lesion noted.	No systemic intervention or radiotherapy at that time due to patient’s performance status.
April 2018	The patient remained in ECOG-4. The patient and family wished to return home.		Discussion held with patient and family. Decision for the patient to return to his community hospital for palliative care.
May 2018	The patient’s condition improved significantly during rehabilitation to ECOG-2.		Patient discharged from community hospital.
June 2018	Patient reassessed by Radiation Oncology.	MRI head and spine: enlargement of pineal tumor. No evidence of drop metastases within spinal column.	
July 2018	Radiation regimen of 54 cGy in 30 fractions to primary tumor and resection cavity. 3600 cGy in 20 fractions to craniospinal axis.		
July 2018	Patient reassessed by Medical Oncology. Discussion held with the patient and family members about the role of systemic therapy with palliative intent.		The patient was provided with information about temozolomide and capecitabine. Systemic therapy planned for post-radiation.
September 2018	Radiotherapy completed. The patient started temozolomide alone based on ECOG, but experienced significant toxicity, resulting in hospital admission.	MRI head and spine: residual tumor slightly decreased in size post-radiotherapy.	Further chemotherapy held.
December 2018	Patient reassessed by Medical Oncology.	MRI head: slight decrease in size of residual tumor with stable edema and hydrocephalus.	No further systemic therapy at this time. Continued surveillance. Referral to Palliative Care for best supportive care.
March, April 2019	Routine follow-ups with Radiation Oncology.	MRI head: stable residual tumor size.	
June 2019	Patient noticed posterior neck swelling.	Biopsy: metastatic disease. MRI cervical spine: at least four enhancing lesions.	Biopsy of neck mass performed.
July 2019	Patient reassessed by Medical Oncology and Radiation Oncology. Planned for radiotherapy to control metastatic disease with possible platinum-based chemotherapy post-radiation.		
August 2019	Patient became unwell before radiotherapy attempted.		No radiation due to decline in performance status. .
September 2019	The patient’s condition deteriorated and was admitted to hospital for palliative care, where he later died.		

*Abbreviations*: *CT* Computed tomography, *IV* Intravenous, *ICP* Intracranial pressure, *ECOG* Eastern Cooperative Oncology Group, *MRI* Magnetic resonance imaging

### Pathology findings

**Fig. 3:**
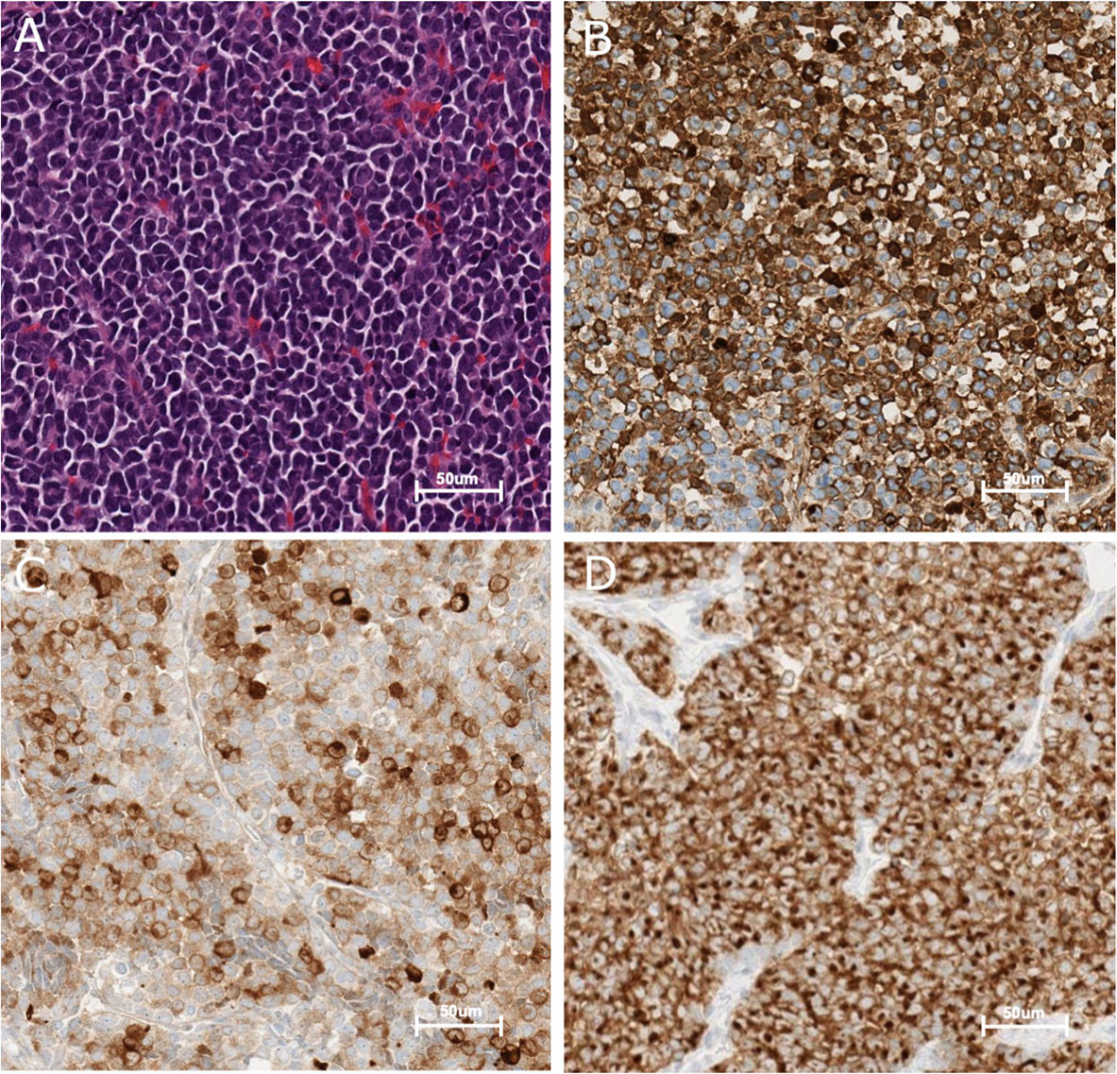
Photomicrographs of the tumor tissue. **A** The tumor consists of uniform round cells arranged in diffuse sheets and trabeculae. Cells contain minimal cytoplasm and indistinct nucleoli. **B** Immunohistochemistry. Tumor cells stain positive for CK 8/18. **C** Immunohistochemistry. Tumor cells stain positive for NSE. **D** Immunohistochemistry. Tumor cells stain positive for synaptophysin

The gross total pineal tumor was soft, tan, and hemorrhagic. Microscopic examination of the biopsied and resected tissues from the pineal region revealed that they were similar in composition, and both included a highly cellular neoplasm composed of primitive-looking round cells with finely dispersed granular chromatin, indistinct nucleoli, and scanty cytoplasm (Fig. 3A). The cells were arranged in diffuse sheets and trabeculae. There were focal papillary arrangements and vague rosettes. Mitotic activity was brisk with 20 per 10 high power fields. Atypical mitotic forms were also seen. Immunohistochemistry was performed and the tumor cells were strongly positive for synaptophysin, neuron specific enolase (NSE), cytokeratin (CK) 8/18, CK AE1/AE3, and CK CAM 5.2 (Fig. 3B, C, D). There was focal positivity for chromogranin, S100, and neurofilament (NF). There was no staining for PTH, epithelial membrane antigen (EMA), CK 7, CK 20, thyroid transcription factor-1 (TTF-1), glial fibrillary acidic protein (GFAP), and microtubule associated protein 2 (MAP2). Integrase interactor 1 (INI1) nuclear staining was intact. The Ki67 index was estimated to be 60–70%.

The slides were scanned using the Leica Aperio AT2 scanner and viewed with the Leica eSlideManager software. The images were acquired at a magnification of 20X and resolution of 0.50uM/pixel. No digital enhancements or adjustments were performed on any of these images.

## Discussion and conclusions

NETs arise from Kulchitsky cells, a type of enterochromaffin cell mostly located in the gastrointestinal and bronchopulmonary tracts, resulting in a propensity for tumours in these locations. However, many epithelial cells and their progenitors can exhibit neuroendocrine differentiation and therefore, although less frequently, NETs can originate in various anatomical regions. In this report, we described a patient with a primary neuroendocrine carcinoma of the pineal gland.

### Diagnostic approach

In this case, the diagnosis of a primary neuroendocrine carcinoma was based on thorough review of pathology and imaging with PET-CT.

#### Pathology

Based on the dual expression of neuroendocrine markers and cytokeratin markers on the resected and biopsied tissue, the diagnosis favoured a neuroendocrine carcinoma. This was supported by histological review by four external pathologists. The World Health Organization (WHO) currently does not include neuroendocrine tumors in its description of pineal tumors [10]. Instead, the WHO describes two main categories of pineal tumours: pineal parenchymal tumours (PPT) [including the pineocytomas, pineal parenchymal tumor of intermediate differentiation, and pineoblastomas] and papillary tumors of the pineal region (PTPR) [10]. Histologically, PPTs are characteristically positive for NSE and synaptophysin, while PTPR demonstrate papillary features and keratin reactivity (CK AE1/AE3, CK 18, CAM 5.2). Intriguingly, there is a reported case of a pineal tumour that was biopsied and subsequently resected at a later date, in which the biopsied tissue was histologically similar to a PPT, while the resected tissue had features of a PTPR [11]. The authors suggest that this phenomenon occurred because the tumor was transitioning from a PPT to a PTPR. It is also feasible that this finding resulted from a sampling error. Another possibility is that this tumor was actually a neuroendocrine carcinoma, as the presence of neuroendocrine markers along with cytokeratin positivity is consistent with a neuroendocrine carcinoma. Our case, along with this case by Cohan et al. highlights the gaps in the current WHO pineal tumor classification guidelines.

#### Diagnostic imaging

The PET/CT showed no primary lesion other than the pineal gland. As mentioned, a possible RCC was incidentally found, but this was unlikely to be the primary site due to the lack of FDG avidity.

### Treatment approach

The patient underwent subtotal resection of the tumor and craniospinal radiation with a plan to administer systemic therapy post-radiation. Initially, the plan was to treat with capecitabine and temozolomide chemotherapy in combination as there is existing evidence demonstrating the effectiveness of this doublet in the treatment of advanced NETs [12]. Furthermore, both agents have shown activity in central nervous system tumours [13, 14]. However, given the patient’s performance status, he received temozolomide alone. Temozolomide was selected because it is an alkylating agent that crosses the blood brain barrier, with a proven efficacy against primary central nervous system tumours, such as gliomas [14]. Platinum-based treatment was ruled out at this point, as the patient was not systemically well enough to receive doublet therapy. Unfortunately, the patient did not tolerate the temozolomide. Treatment with capecitabine and temozolomide in combination would have provided valuable insight into the efficacy and tolerability of treating rare intracranial NETs. The only systemic therapy regimens previously published for this type of intracranial tumour were cisplatin and etoposide with and without sunitinib, or single agent temozolomide.

**Fig. 4:**
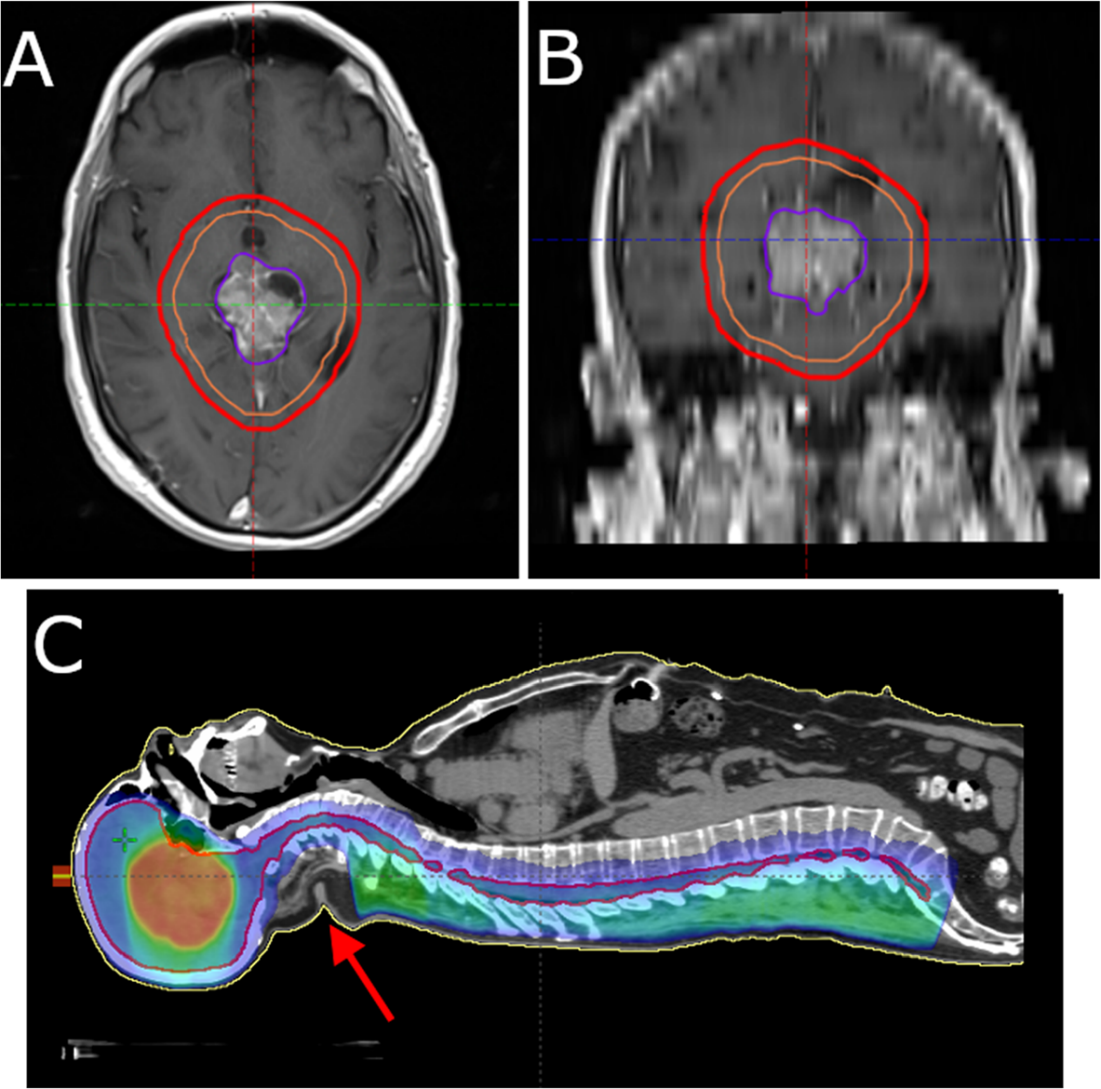
Images demonstrating the radiation field. **A** Axial head view, with 5400 cGy volume. **B** Coronal head view, with 5400 cGy volume. **C** Sagittal body view with 3600 cGy volume and dose colourwash. The arrow indicates the low dose region and surgical tract where the disease recurred

From a Radiation Oncology perspective, this was a remarkable case given the pattern of disease recurrence. As this disease entity is exceedingly rare, there were no pre-existing standards for radiotherapy. Knowing that this was a high-grade tumor, we treated the patient with craniospinal irradiation, which is standard for pineoblastomas, another high-grade pineal tumor. The treatment focuses on the craniospinal axis, along with a boost to the intracranial cavity, and does not involve radiation to the surgical scar and dermis (Fig. 4A, B, C). Unexpectedly, the marginal failure involved the soft tissues in the surgical site outside of the radiation field and the patient developed lymphadenopathy in his neck. In the future, we would strongly consider including the surgical tract all the way to the epidermis in the 3600 cGy volume to prevent a marginal failure. It is unclear whether including lymph node levels in the neck would prevent a locoregional failure.

### Intracranial primary neuroendocrine tumors in the literature

There are few instances in the literature of intracranial NETs, either as primary occurrences or metastases from other sites [2]. We identified eight published case reports of primary intracranial NETs [2–9] (Table 2) including primaries near the pituitary, along the meninges, in the pineal gland, and intraventricularly [2–9]. Patients in these case reports either underwent surgical resection or excisional biopsy only [2–9]. In four of these cases, treatment included systemic therapy and three of these patients also received radiation therapy [2, 3, 8, 9]. In the previously reported case of a primary neuroendocrine tumor of the pineal gland, a chemotherapy regimen of cisplatin, etoposide, and sunitinib was administered, along with craniospinal radiation, and the patient died 26 months following their diagnosis [2]. In the case of a primary intraventricular neuroendocrine carcinoma, cisplatin and etoposide were used as systemic therapy, along with intensity-modulated radiation therapy [8]. That patient tolerated therapy and was followed for 5 years, experiencing no recurrence until 10 years later [8]. During treatment of recurrence, the patient experienced worsening issues related to her ventriculoperitoneal shunt secondary to hydrocephaly and died due to complications [8]. This highlights the importance of ongoing and long-term surveillance post-remission in cases of primary intracranial NETs. In one of these cases, multiple primary intracranial NETs were found in a single patient. This patient underwent surgical resection of the largest lesion, followed by whole brain and tumor bed radiation, and systemic therapy using temozolomide resulting in decrease in tumor volume and no recurrence at the 10-month follow-up [9].

**Table 2:** Published cases of intracranial neuroendocrine tumors of primary origin

Source	Intracranial Location	Age (y.)	Sex	Treatment	Overall survival	Cause of death
Hakar et al.	Pineal parenchyma	35	Female	Subtotal resection Cisplatin, etoposide, sunitinib Craniospinal irradiation	Died 26 months following diagnosis	Cancer progression
Liu et al.	Sella/hypothalamic	39	Female	Resection	Died three months following diagnosis	Cancer progression, too unwell to receive radiotherapy
Liu et al.	Anterior cranial fossa	40	Female	Resection	At time of publication, patient alive, but undisclosed timeline	-
Reed et al.	Intraventricular, third ventricle	34	Female	Resection Intensity modulated radiation therapy Cisplatin and etoposide	Followed for five years with no recurrence Recurrence 10 years after initial diagnosis, died 2 months after	Complications of hydrocephalus secondary to recurrence
Ibrahim et al.	Skull base, level of jugular foramen	29	Female	Monthly somatostatin injections Patient declined radiotherapy	Followed for one year with stable disease	-
Hood et al.	Left cavernous sinus	61	Female	Subtotal resection Fractionated external beam radiation therapy	Followed for 7.5 years with stable disease	-
Deshaies et al.	Dural-based, compressing right frontal lobe	79	Female	Resection Octreotide	Died 6 weeks after diagnosis	Unknown complications
Cao et al.	Multiple lesions, left parietal convexity, right occipital parietal convexity, left frontal convexity, left cerebral falx, right temporal lobe	56	Female	Resection of three lesions Whole brain and tumor bed radiotherapy Temozolomide with recombinant human endostatin	Followed for 10 months with stable disease	-
Porter et al.	Right cerebellopontine angle of posterior fossa	62	Male	Subtotal resection	At time of publication, 5-year survival	-
Our study	Pineal parenchyma	53	Male	Subtotal resection Temozolomide Craniospinal irradiation	Died 18 months after diagnosis	Cancer progression

### Metastatic intracranial neuroendocrine tumors

Metastatic intracranial NETs are also quite uncommon. There are few reported cases of brain metastases occurring in patients with NETs of non-lung, gastroenteropancreatic, or bronchopulmonary origin, with an estimated incidence of 1.5–5% [15]. In one study of patients with NETs and brain metastases, lung was noted to be the most frequent primary disease site [16]. As such, there are recommendations for MRI of the head of part of the metastatic workup in patients with primary carcinoma of the lung [15]. In patients with single brain metastases, surgery is indicated with a palliative role [15]. Systemic therapy is typically chosen based on the tumor origin and biology [15]. In those with multiple intracranial metastases, external beam irradiation is indicated and can be combined with surgery [15]. Palliative radiation was the most common treatment type found in a cohort of 51 patients with secondary intracranial neuroendocrine disease [16]. In patients with brain metastases, median survival is less than 10 months with a 1-year survival rate of < 40% [15]. Given the difference in prognosis and treatment indications between primary and secondary disease, there is value in performing a thorough metastatic work-up to establish tumor origin.

### Patient perspective

Unfortunately, the patient died before being able to write about his experience with this disease and his care. His partner had provided written consent for publication.

## Conclusion

In conclusion, intracranial NETs are an uncommon entity and those arising in the pineal gland are exceedingly rare. There is limited evidence pertaining to the care of affected patients published in the literature. We report on a case of NET of the pineal gland to contribute to research in this area and ultimately improve health care delivery for patients with these tumours. Although uncommon, NETs should be considered in the differential diagnosis of intracranial lesions.

## Data Availability

Not applicable.

## Abbreviations

NET: Neuroendocrine tumor
CgA: Chromogranin A
NSE: Neuron specific enolase
CSF: Cerebrospinal fluid
CT: Computed tomography
MRI: Magnetic resonance imaging
EVD: External ventricular drain
ETV: Endoscopic third ventriculostomy
PET/CT: Positron emission tomography-computed tomography
FDG: Fluorodeoxyglucose
RCC: Renal cell carcinoma
PTH: Parathyroid hormone
ECOG: Eastern Cooperative Oncology Group
CK: Cytokeratin
NF: Neurofilament
EMA: Epithelial membrane antigen
TTF-1: Thyroid transcription factor 1
GFAP: Glial fibrillary acidic protein
MAP 2: Microtubule associated protein 2
INI1: Integrase interactor 1
PPT: Parenchymal pineal tumor
PTPR: Papillary tumor of pineal region
